# Microgravity-Induced Fluid Shift and Ophthalmic Changes

**DOI:** 10.3390/life4040621

**Published:** 2014-11-07

**Authors:** Emily S. Nelson, Lealem Mulugeta, Jerry G. Myers

**Affiliations:** 1 NASA Glenn Research Center, 21000 Brookpark Rd., Cleveland, OH 44135, USA; 2 Universities Space Research Association, Division of Space Life Sciences, 3600 Bay Area Boulevard, Houston, TX 77058, USA; E-Mail: lealem.mulugeta@nasa.gov; 3 NASA Glenn Research Center, 21000 Brookpark Rd., Cleveland, OH 44135, USA; E-Mail: Jerry.G.Myers@nasa.gov

**Keywords:** microgravity, aerospace medicine, visual impairment, intracranial pressure, cephalic fluid shift, gravitational physiology

## Abstract

Although changes to visual acuity in spaceflight have been observed in some astronauts since the early days of the space program, the impact to the crew was considered minor. Since that time, missions to the International Space Station have extended the typical duration of time spent in microgravity from a few days or weeks to many months. This has been accompanied by the emergence of a variety of ophthalmic pathologies in a significant proportion of long-duration crewmembers, including globe flattening, choroidal folding, optic disc edema, and optic nerve kinking, among others. The clinical findings of affected astronauts are reminiscent of terrestrial pathologies such as idiopathic intracranial hypertension that are characterized by high intracranial pressure. As a result, NASA has placed an emphasis on determining the relevant factors and their interactions that are responsible for detrimental ophthalmic response to space. This article will describe the Visual Impairment and Intracranial Pressure syndrome, link it to key factors in physiological adaptation to the microgravity environment, particularly a cephalad shifting of bodily fluids, and discuss the implications for ocular biomechanics and physiological function in long-duration spaceflight.

## 1. Introduction

In order to reach low earth orbit on the Space Shuttle, astronauts were seated on top of a carefully designed but monumentally powerful explosion that could lift a mass of over 4 million pounds (about 1.8 million kg) up into orbit. As they ascended, they were exposed to a hypergravitational field that, at times, reached up to three times earth gravity. After about 9 min, the main engines shut off. At this point, the Shuttle was circling around the earth at an altitude of about 200 miles (320 km) above the earth surface and at an orbital velocity of about 17,500 miles per hour (~28,000 km/h). Under these conditions, the apparent gravitational force on the astronauts was negligible, and they experienced the sensation of weightlessness (see Section 3.1). Within moments, they exhibited a marked swelling in the tissues of their head and neck, and soon their waistlines and legs became substantially slimmer while their chest expanded (Section 3.2). After the gross volume redistribution upon entry into orbit, bodily fluids continued to refine their distribution throughout the body, but it retained its essential character of greatly increased volume in the upper regions of the body at the expense of the lower regions. After a couple of weeks, the body reached a homeostatic distribution that was retained throughout their time in space (Section 3.2) [1] and it was reversible upon their return to earth. The details of the launch conditions may change somewhat from that described here, depending on the carrier, such as the Progress rocket or commercial vehicles that are currently under development. Nevertheless, the general features of microgravity-induced fluid shift remain the same for any human being in low earth orbit.

The record for longest mission duration on orbit is held by the Russian space station Mir, which hosted individuals for up to 438 days on a single mission throughout its lifespan from 1986 to 2001. Even today, Soviet/Russian cosmonauts still hold the record for the maximum number of person-days in space. Crewed missions conducted research on the US Skylab for up to 3 months in 1973 to 1974, and provided some of the most comprehensive data on fluid shift to date. The pool of astronauts who have served on the US Space Shuttle and the International Space Station (ISS) is much larger than earlier ventures into low earth orbit. Typical mission duration for astronauts expanded from about 10 to 16 days in the Shuttle era to a routine span of ~six months on the ISS. NASA is currently planning one-year missions on the ISS to commence in 2015. At present, 35 countries have participated in human spaceflight. The combined experience of over 500 individuals continues to provide us with a richer understanding of human response to living and working in a spacecraft environment.

Physiological conditions of concern that accompany spaceflight are a propensity toward bone loss [2], muscle atrophy [3], postflight orthostatic intolerance [4,5], and the development of kidney stones [6], among others. This article is concerned specifically with microgravity-induced ophthalmic changes (see Section 2). Since ophthalmic pathologies appear to be linked to increased duration in space (Section 2), it becomes even more crucial to understand the root causes and the patterns of development of ophthalmic neuropathology resulting from exposure to microgravity on the ISS. The sequence of events that lead to ophthalmic change in space is not yet understood, but a prime candidate for the initiating event is the profound impact of the cephalic fluid shift. Consequently, this article explores the fluid mechanics and biophysics of fluid shift in space in some depth (Section 3). The mechanisms by which fluid shift and other factors in the spacecraft environment could affect ocular function are discussed in Section 4. Many excellent review papers are available for a broader understanding of human physiological response to space, e.g., [5,7,8,9,10,11,12,13] and clinical observations on the ophthalmic changes in astronauts, e.g., [7,14,15,16].

## 2. The Visual Impairment and Intracranial Pressure (VIIP) Syndrome

Indications regarding the influence of the microgravity environment on visual performance were present as early as the Mercury missions when astronauts reported changes to their eyesight during flight [17,18]. Although visual acuity tests during Gemini V and Gemini VII showed no statistically significant change in visual capability, vision system physiology investigations during the Apollo program revealed an increase in Intraocular Pressure (*IOP*) [17,18]. This was further corroborated by Spacelab experiments that showed mean *IOP* rise of 20%–25% during flight, and a decrease below baseline levels postflight [17,18]. Similar investigations were conducted during Shuttle missions and in microgravity analog environments that also confirmed the *IOP* increase during acute exposure [7,19,20,21,22,23,24]. Overall, the ophthalmic changes were minor or transient, without other symptoms or clinical findings, and were not uncommon in the broader 40 to 50 year-old population. However, it was recently discovered that a significant proportion of the astronauts who have participated in long-duration missions are experiencing noticeable and persistent ophthalmic changes and pathological conditions [7,16].

Of the 36 astronauts from the United States who have flown on long-duration missions of approximately six months aboard the International Space Station, 15 crewmembers (42.7%) have been diagnosed with the symptoms of the Visual Impairment and Intracranial Pressure (VIIP) syndrome, while 19 others are still undergoing medical evaluation. Only two are confirmed to be without symptoms [25]. The changes observed postflight included decreased *IOP*, the presence of choroidal folds, posterior globe flattening, a tortuous (kinked) optic nerve and elevated cerebrospinal fluid (CSF) pressure [15,16]. Some astronauts experienced temporary ophthalmic changes, while several others maintained persistent changes after returning from flight. One crewmember in particular has been reported to exhibit refractive decrements for more than five years after returning to earth [16].

Given that many of these astronauts present signs that are similar to those observed in idiopathic intracranial hypertension (IIH) patients on earth, it has been hypothesized that these changes may be caused by microgravity-induced cephalic fluid shift (see [7,16]). The reasoning is that the increased intracranial fluid volume could produce elevated Intracranial Pressure (*ICP*) that could in turn cause ophthalmic changes like optic disc edema [26,27]. Other environmental factors such as the elevated levels of carbon dioxide on ISS may further exacerbate the propensity toward VIIP development (see Section 4.2). Subject matter experts have suggested that some crewmembers may be more susceptible than others due to interindividual factors such as genetics, anatomical features or physical fitness [7].

Although the specific symptoms of all 15 crewmembers have not yet been published, their conditions have been classified under NASA’s clinical practice guidelines (CPG) [7,25] as noted in Table 1. It is interesting to note that five of the affected crewmembers (33% of the affected) fall under CPG class 3 and 4, the most severe classification levels. These same five crewmembers also exhibited the lowest *IOP*s both before and after flight, and experienced the greatest drop in pre- to postflight *IOP*, which was nearly 50% more than any other classification [7]. The pre- and postflight mean *IOP* values were 13.2 and 11.8 mmHg respectively, while normal *IOP* levels in the general healthy population range from 10 to 20 mmHg. Large-scale studies find the mean *IOP* to be in the range of 14–15 mmHg for healthy subjects on earth [28,29,30].

**Table 1:** Clinical Practice Guidelines classifications of all 36 long-duration spaceflight crewmembers from the United States. Data from [25].

CPG Class	Definition	No. of Affected Crewmembers
Non Cases	<0.50 diopter cycloplegic refractive change No evidence of papilledema, nerve sheath distention, choroidal folds, globe flattening, scotoma or cotton wool spots compared to baseline	2
1	(repeat Optical Coherence Tomography (OCT) and visual acuity in 6 weeks) ≥0.50 diopter cycloplegic refractive change and/or cotton wool spot No evidence of papilledema, nerve sheath distention, choroidal folds, globe flattening, scotoma compared to baseline CSF opening pressure (if measured) ≤ 25 cmH_2_O	2
2	(repeat OCT, cycloplegic refraction, fundus exam and threshold visual field every four to six weeks x six months, repeat Magnetic Resonance Imaging (MRI) in six months) ≥0.50 diopter cycloplegic refractive changes or cotton wool spot Choroidal folds and/or optic nerve sheath distension and/or globe flattening and/or scotoma No evidence of papilledema CSF opening pressure ≤ 25 cm H_2_O (if measured)	8
3	(repeat OCT, cycloplegic refraction, fundus exam and threshold visual field every four to six weeks x six months, repeat MRI in six months) ≥0.50 diopter cycloplegic refractive changes and/or cotton wool spot Optic nerve sheath distension, and/or globe flattening and/or choroidal folds and/or scotoma Papilledema of Grade 0–2 CSF opening pressure ≤ 25 cm H_2_O	1
4	(institute treatment protocol as per CPG) ≥0.50 diopter cycloplegic refractive changes and/or cotton wool spot Optic nerve sheath distension, and/or globe flattening and/or choroidal folds and/or scotoma Papilledema Grade 2 or above. Presenting symptoms of new headache, pulsatile tinnitus and/or transient visual obscurations CSF opening pressure >25 cm H_2_O	4
Unclassified	Too little evidence at present for definitive classification Early ISS flyers with no, or limited testing	19

Out of the affected 15 crewmembers, Mader *et al*. [16] have reported clinical findings of ophthalmic changes for seven crewmembers, which are summarized in Table 2. Of the seven, three did not report any changes to visual acuity during flight. The others initially reported a visual acuity change at three weeks to three months into flight. The affected individuals to date have all been men between 45 and 55 years of age. No women have been identified as affected by VIIP at this time. However, the number of female astronauts who have flown on long-duration missions is currently too small to assume that women are less susceptible to the VIIP syndrome than their male counterparts.

**Table 2:** Summary of ophthalmic changes from seven affected long-duration mission crewmembers. Data from [7,16].

Ophthalmic Condition	Total Affected
Optic nerve sheath distension	6/7 (86%)
Nerve fiber layer thickening	6/7 (86%)
Optic disc edema	5/7 (71%)
Posterior globe flattening	5/7 (71%)
Hyperopic shift in one or both eyes by >+0.50 diopters	5/7 (71%)
Choroidal folds	4/7 (57%)
Elevated postflight CSF pressure (indicative of increased *ICP*)	4/7 (57%)
Cotton wool spots	3/7 (43%)
Decreased intraocular pressure (*IOP*) postflight	3/7 (43%)
Tortuous optic nerve	2/7 (29%)

Mader *et al*. [16] also conducted a retrospective survey of 300 Shuttle astronauts and found that approximately 23% had complaints of near-vision degradation, and 11% had a documented record of postflight hyperopic shift in their vision. This suggests that the genesis of VIIP may in fact begin within the two-week timeframe of a typical Shuttle mission.

**Figure 1:**
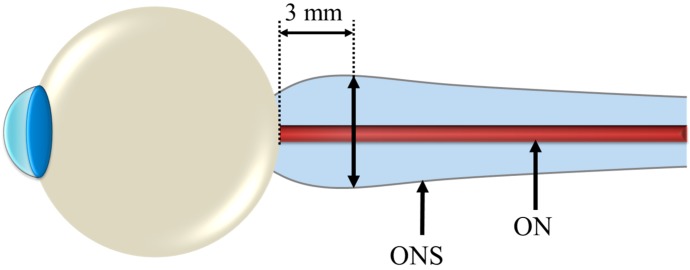
Reference location of Optic Nerve Sheath Diameter (ONSD) measurement for determining risk of elevated Intracranial Pressure (*ICP*).

Kramer *et al*. [15] performed follow-on quantitative and qualitative magnetic resonance (MR) analyses of the orbital and intracranial structures of 27 astronauts, irrespective of mission duration or previous clinical findings. The results of this study confirmed the findings of Mader *et al.* [16] regarding optic nerve sheath (ONS) distention, posterior globe flattening, optic disc protrusion (indicative of edema), increased optic nerve diameter (OND), and increased tortuosity of the optic nerve (ON). In addition, they discovered three long-duration crewmembers and one short-duration astronaut who presented moderate or greater concavity of the pituitary gland with posterior stalk displacement, which can be a strong indicator of intracranial hypertension. Moreover, based on findings from a study that showed a 90% probability of intracranial hypertension for optic nerve sheath diameter (ONSD) greater than 5.82 mm at 3 mm posterior to the globe [31] (illustrated in Figure 1), they identified 14 out of the 27 astronauts who may be at risk of elevated *ICP*. Nine of them were among the long-duration crewmembers known to be affected by VIIP.

**Figure 2:**
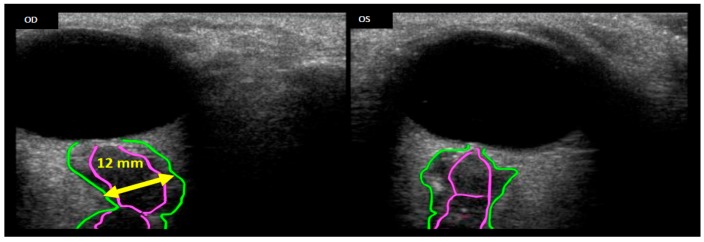
Remotely guided on-orbit ultrasound images showing the OD (right eye) Optic Nerve Sheath Diameter (ONSD, green outline) distended to approximately 12 mm, thus suggesting increased Intracranial Pressure (*ICP*). Comparing the optic nerve (pink outline) of the right eye with the left (OS) eye, a kink can clearly be seen in the OD optic nerve. Source: NASA Longitudinal Study of Astronaut Health.

**Figure 3:**
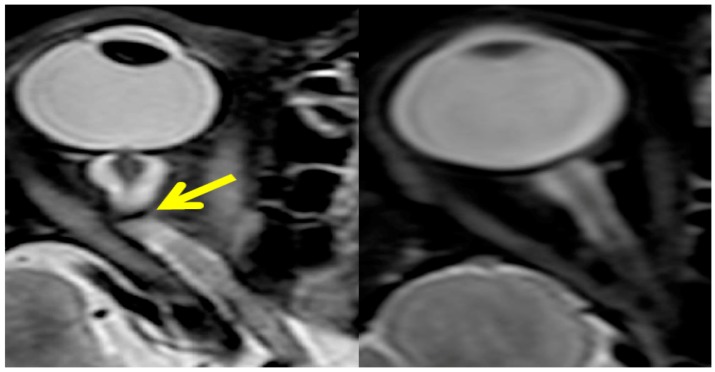
The postflight magnetic resonance image on the left shows that optic nerve kinking is present, while the control orbit on the right does not exhibit any tortuosity in the nerve. Source: National Aeronautics and Space Administration.

**Figure 4:**
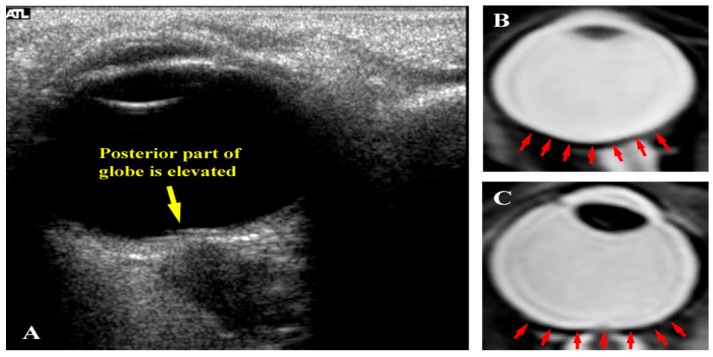
(**a**) Inflight ultrasound of a globe showing flattening of the posterior globe; (**b**) Magnetic resonance image (MRI) of a normal globe before long-duration spaceflight; and (**c**) MRI of a globe after a long-duration flight exhibiting posterior flattening. Source: NASA Longitudinal Study of Astronaut Health.

Seven astronauts were identified with posterior globe flattening with mean ONSD of 7.2 + 1.5 mm, six of whom were long-duration flyers. Four others, who had a mean ONSD of 7.5 ± 1.1 mm, exhibited ON kinking. Given that posterior globe flattening, ON kinking and ONSD distension have been associated with IIH [32,33,34,35], this further strengthens the hypothesis of elevated *ICP* in flight [7,16]. Figure 2, Figure 3 and Figure 4 show inflight ultrasound and postflight MR images of the globe, and the ON and ONS exhibit clear indications of these symptoms.

These findings are not conclusive. Kramer *et al*. [15] note that their work is limited by the fact that postflight MR images were acquired well after landing (months to years), as well as the lack of a control astronaut group without flight experience, concurrent CSF pressure or *IOP* measurements, and baseline MR venograms to exclude dural sinus stenosis found in association with IIH. The authors note that their quantitative analysis technique for ONSD measurements may be biased to larger diameter measurements than previously reported [15]. Therefore, further work is required to verify whether or not intracranial hypertension is an etiological factor. Until then, the different conditions should not be diagnosed or interpreted to be the same as IIH. For this reason, terms like “papilledema” are not used to refer to the optic disc swelling observed in VIIP syndrome. Papilledema, by definition, is swelling of the optic disc due to elevated *ICP*. In other words, even if *ICP* is elevated, it must be shown to be the primary cause of optic disc swelling, and not due to other factors such as ocular hypotony (low *IOP*). Under both conditions the optic nerve head can swell as a result of increased translaminar pressure across the optic nerve head, which can lead to the risk of optic nerve damage that can adversely impact vision [7]. It should be emphasized that there is no definitive evidence of ocular hypotony or elevated ICP in the affected astronaut population at this time.

Overall, the constellation of symptoms associated with VIIP syndrome is quite peculiar because many of the symptoms manifest similar to what would be observed in terrestrial clinical conditions such as IIH, hydrocephalus, and ocular and orbital tumors. However, astronauts are healthy individuals who have no known history for any of these or other similar medical conditions. Moreover, while the signs of VIIP syndrome are often compared to those observed in IIH patients, none of the affected crewmembers have postflight complaints of headaches, double vision, pulsatile tinnitus, or transient visual obstruction [16], which are often associated with IIH [36]. This is further complicated by the fact that, even though IIH is a well-characterized clinical problem in terrestrial medicine, the root causes of the disease are not well understood (*i.e.*, idiopathic).

**Figure 5:**
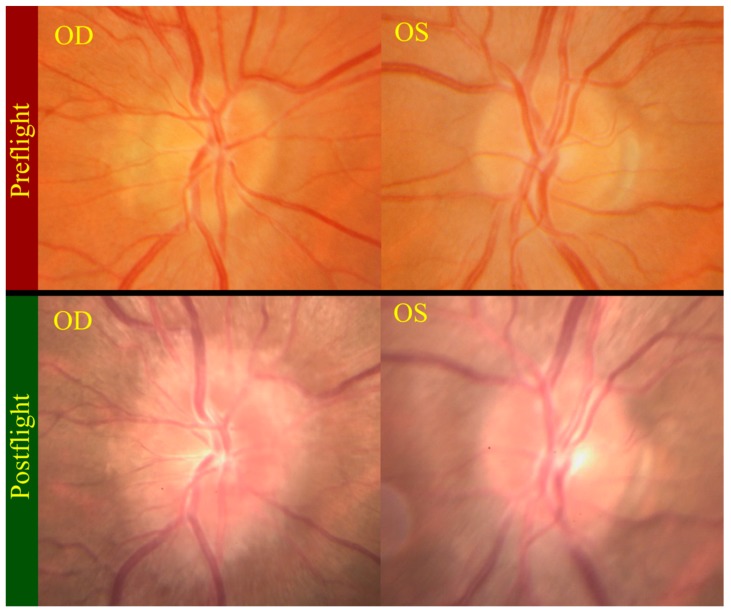
Postflight fundus examination photos (bottom) show grade 3 and grade 1 optic disc edema in the right (OD) and left (OS) optic discs respectively. The top photos show the preflight fundus examination of normal optic discs. Source: NASA Longitudinal Study of Astronaut Health.

Another unusual aspect of VIIP syndrome is that not all crewmembers experience visual changes even when some of the other symptoms they experience may be quite pronounced. In one case, the crewmember did not report any symptoms during or after flight. Postflight medical examinations, however, revealed that the crewmember had the worst case of optic disc edema (swollen optic disc) (Figure 5), a small hemorrhage inferior to the optic disc of the right eye, nerve fiber layer thickening and elevated CSF pressure as obtained by lumbar puncture. On the other hand, the crewmember showed no signs of choroidal folds, posterior globe flattening or hyperopic shift. None of the other crewmembers were found to have small hemorrhages similar to this subject [7,16].

Persistent posterior globe flattening is another finding that has many experts puzzled. Under terrestrial conditions, posterior globe flattening has been known to be induced by intracranial hypertension [32,33]. Following treatment for IIH, there do not appear to be any studies that verify whether or not globe flattening persists or is resolved. Therefore, it is not certain whether or not localized increased pressure behind the optic disc alone can cause permanent posterior globe flattening. Such a risk has been noted by Wu *et al*. [37], who reported on a patient with an intraocular tumor located behind the globe that was suspected to have been present for at least five years. This prolonged pressure on the back of the eye apparently caused a posterior flattening of the globe and hyperopic shift by +3 diopters, which persisted even 18 months after the intraocular mass was removed. The author suggests that failure to resolve the anatomical and refractive changes suggests the possibility of remodeling of the sclera due to chronic compression.

**Figure 6:**
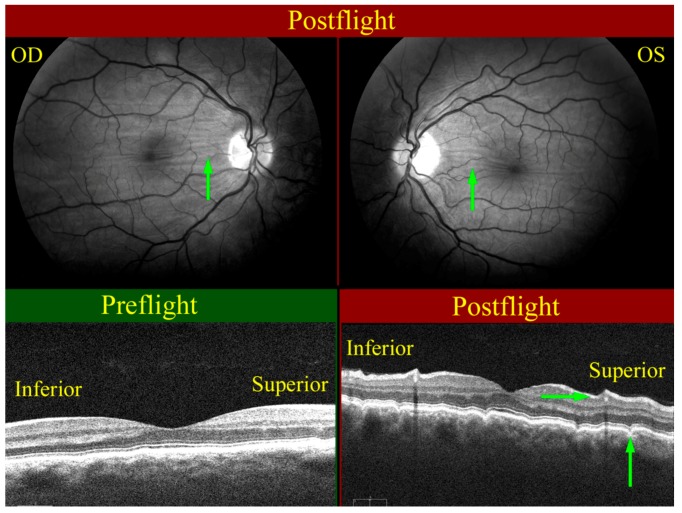
Choroidal folds (green arrows) as seen on the right (OD, upper left), and left (OS, upper right) globes. The wavy pattern (highlighted by green arrows) in the choroidal/retinal layer shown in the OCT image taken post-flight (bottom right) relative to the pre-flight OCT image (bottom left) exhibits the presence of choroidal folds. Source: NASA Longitudinal Study of Astronaut Health.

As noted earlier, many crewmembers also get choroidal folds (Figure 6), which are generally described as parallel alternating bright (peaks) and dark (troughs) streaks that are typically found at the posterior pole region of the globe. In terrestrial medicine, choroidal folds are known to be associated with a wide variety of pathological conditions, such as papilledema, ocular and orbital tumors, flattening of the posterior pole, and choroidal thickening due to vascular engorgement, to name a few. They may also be induced by surgical procedures that result in post-operative reduction of *IOP*. However, in a majority of the cases, they are idiopathic in nature and can be associated with benign ophthalmic conditions such as hyperopia [38,39,40]. Therefore, given that the cause of choroidal folds is not well understood under terrestrial conditions where there is greater breadth and depth of understanding of human physiology, there is even greater uncertainty regarding pathophysiology of its development under spaceflight conditions and the long-term implications on the health of astronauts. It is well recognized, however, that the pattern and orientation of the folds can give insight into the contributing factors and the potential for adverse health outcomes [41]. For example, it has been postulated that radial choroidal folds are associated with choroidal neovascularization, which can cause scotoma and vision loss.

**Figure 7:**
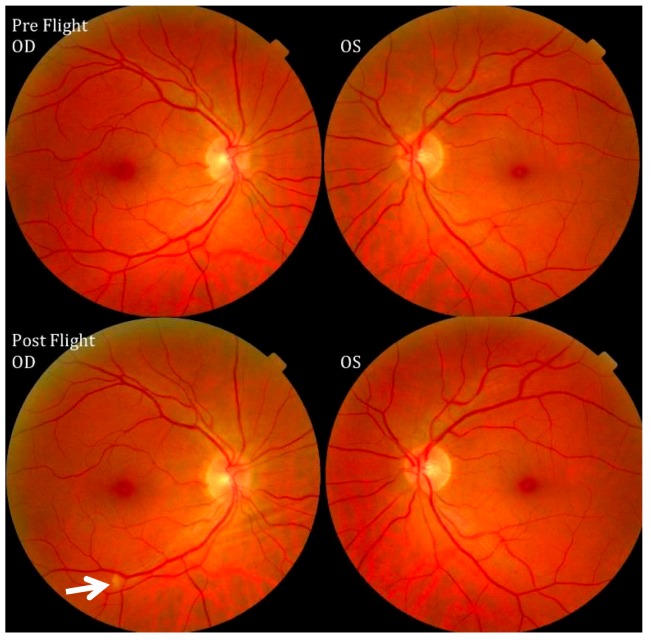
Pre- and postflight fundus examination images of the first Visual Impairment and Intracranial Pressure (VIIP) case. As identified by the white arrow in the right eye, a single cotton-wool spot was discovered. Source: National Aeronautics and Space Administration.

Cotton-wool spots (CWSs) have also been discovered in some crewmembers, as noted by Mader *et al*. [16] and illustrated in Figure 7. CWSs generally appear as spots with whitish-grey and fluffy deposits exhibiting frayed edges. They also lie superficially as opaque swellings in the retina as acute lesions that can occur in various diseases involving the retinal vascular system. The presence of a CWS is an indication of serious vascular damage [7], and the presence of a CWS should be treated as an important clinical sign [42].

At this time, the root cause(s) and subsequent development of the VIIP syndrome are not well understood, and the challenges of working in the novel environment of space makes it difficult acquire sufficient data to draw meaningful conclusions regarding its pathophysiology. Many of the terrestrial medical conditions that are analogous to VIIP also seem to be idiopathic in nature. This lack of clarity results in greater uncertainty regarding the causal mechanism of this unique physiological condition observed in astronauts. Moreover, given that the symptoms have been noted only in a portion of the astronaut population with widely varying combinations and severity levels, there may well be variable physiological response to the microgravity environment [15]. Therefore, dedicated research is needed to identify the relevant disease pathways that could result in adverse impact to astronaut vision and neurological health, as well as addressing important questions regarding the vast knowledge gaps that exist in related terrestrial diseases.

## 3. Fluid Redistribution in Microgravity

Perhaps the most obvious and profound physiological response and adaptation to the microgravity environment is the redistribution of fluid and tissues of the body, which begins as a substantial volume of fluid, primarily from the legs, moves headward upon reaching low-earth orbit [7,43,44]. The gross spatial redistribution occurs rapidly [45,46], but the fluid volume continues to rearrange by moving into various tissues, e.g., interstitial spaces, over a longer period of time [43] as a result of biophysical and biochemical inducement. Sequestration into the interstitial spaces likely contributes to many physiological and anatomical changes, including the well-documented swollen facial tissues and congested sinus passages in early flight [10,47]. In this section, we describe the physical and physiological phenomena that are associated with fluid redistribution.

### 3.1. Reduced Gravity Environments

Gravitational acceleration at the earth’s surface is nominally 9.8 m/s^2^, denoted as *g_e_*. Other accelerations also affect physical systems in gravity-like ways, such as the human body during rapid deceleration of a car. As a fighter plane pulls up tightly, the vehicle acceleration adds to the background gravity so that the fighter pilot may experience a net acceleration of order 10 *g_e_*. The pilot’s body responds to the net acceleration, which includes gravity as well as any other accelerations that are present.

When the International Space Station orbits the earth at 250 miles (402 km) above the earth’s surface, the local gravitational acceleration is only about 7% less than at sea level. However, the additive effect of forces generated by orbital mechanics combine with gravity to create a near-weightless environment [48].

We loosely refer to the reduced acceleration environment on an orbiting vehicle as “microgravity”. In a literal sense, microgravity means 1 × 10^−6^ *g_e_* or 1 μg, and in fact the steady background acceleration may go as low as a few μg at particular locations and times on the ISS [49]. However, the residual acceleration environment is more complex than that: thruster firings for attitude adjustment, astronaut exercise, equipment operation and other sources can directly inject transient, multidirectional, multifrequency components into the net acceleration or they may affect the residual acceleration indirectly by exciting vehicle structural modes [48]. The human body may feel weightless while hardware that includes fluid handling may be quite sensitive to the low-level vibratory accelerations [48]. For many practical problems, however, the effects of the residual acceleration can be ignored. To study such problems, payloads can be dropped into drop towers to achieve a few seconds of microgravity, which is long enough to study some problems in microgravity fluid physics and combustion [50]. For longer periods of reduced gravity, NASA flies airplanes in a parabolic trajectory [50]. As the vehicle rounds the peak altitude, the local acceleration can be as low as 10–100 μg for about 15–20 s. As the plane pulls up, the local net acceleration ramps up to roughly 2*g_e_*. On a typical flight campaign, a set of 30 to 40 parabolas are flown sequentially for repeated measurement opportunities. Unfortunately, these time scales can only study the most acute physiological responses to microgravity [14,20,51,52,53,54,55].

### 3.2. Simple Physical Systems

By definition, a fluid is a substance that deforms when a shear force is applied [56]. Both liquids and gases are fluids, and all fluids are affected by gravity [57]. Newtonian fluids, such as water or honey, are well-behaved and deform directly in proportion to the magnitude of the shear force. The proportionality constant is the fluid viscosity. Blood is a non-Newtonian fluid, but the non-Newtonian effects can be neglected in some situations, such as high-shear flow through major arteries [58]. Deformable solids are also subject to gravity, e.g., gels change shape due to gravitational forces [59]. In the human body, gravitational changes can result in a shift in the position of major organs, and in some cases, there may be a change in organ shape [60,61].

Our perception of weight (*W*) comes from gravity (*g*) acting on mass (*m*), which can be expressed mathematically as *W = mg*. When a body in a uniform gravitational field is opposed by a reaction force, as occurs when standing on the ground, the gravity force and ground reaction force are in equilibrium. This reaction force is not uniform across the body and thus acts on each part of a body in a relative fashion resulting in conditions such as hydrostatic pressure gradients. Conversely in free fall, a uniform gravitational field acts on each part of the body equally in the absence of any other opposing reaction forces such as drag. Although the body is accelerating, by Newton’s famous analysis, this induces relative weightlessness within the body, similar to a truly zero gravitational field. Thus, a body in free fall has a relative experience of zero gravity [48]. This process is slightly different for orbital spacecraft such as ISS, whose angular velocity and apparent centripetal forces create an environment that is essentially weightless. In effect, the spacecraft is falling around the earth [48].

**Figure 8:**
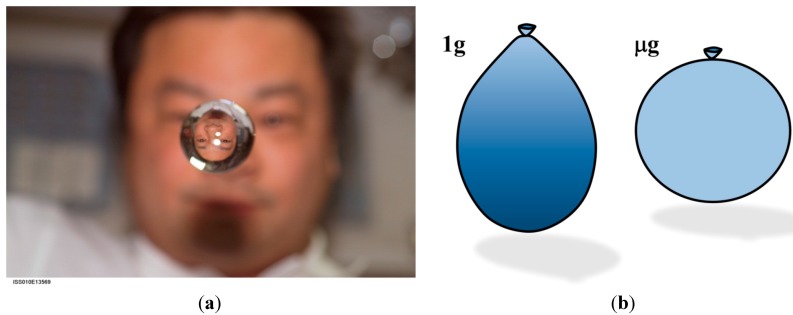
(**a**) Astronaut Leroy Chiao observes his reflected and refracted image in a spherical droplet of water onboard the ISS. Source: National Aeronautics and Astronautics Association [66]; (**b**) The equilibrium shapes for a water-filled balloon in 1 g and microgravity (μg).

Newton’s law of conservation of momentum illuminates the principle that it is the sum of the forces (*i.e.*, the net force) acting on a mass that governs its response [57]. When strong forces such as hydrostatic pressure are (nearly) eliminated in microgravity, weaker forces become dominant and govern fluid behavior [48]. These forces include surface tension, which is a measure of the attractive force between molecules in a fluid [62]. When confronted with a different fluid, fluids with high surface tension will tend to form an interface with their neighbors to maintain their cohesion. An example of such a binary fluid system is oil and water. On earth, the shape of a water droplet is governed by both gravity and surface tension [62]. In space, the equilibrium shape of a small volume of water is spherical due to surface tension acting without the interference of gravity [63,64], as shown in Figure 8a.

If the water is bound by a membrane with uniform elasticity, such as a spherical balloon, then the equilibrium shape in 1 g is roughly egg-shaped due to the combination of hydrostatic pressure forces and the confining force of the membrane, as shown in Figure 8b. Along any vertical line, the local hydrostatic pressure difference between any two points within a continuous region of fluid is equivalent to the weight per unit area of the fluid column between them. It can be quantified by ρ*gh*, where ρ is fluid density, *g* is the gravitational acceleration and *h* is the height of the fluid column [57]. Along the balloon’s centerline, the hydrostatic pressure is at a maximum at the bottom of the balloon and decreases with elevation to zero at the upper tip of the fluid within the balloon. Upon entry into microgravity, the hydrostatic pressure is abruptly removed throughout the fluid volume, and the system is driven to become spherical, similar to the unconfined blob of water. In other words, the microgravity environment drives a fluid system toward symmetry about its center of mass, to the extent that its confining boundaries allow it to do so. This change in shape occurs very rapidly, within a fraction of a second, see, e.g. the shapes of water balloons and unconfined volumes of water in parabolic flight and on ISS [65].

Clearly, fluid redistribution in the human body when exposed to a low-gravity environment is far more complex than that of a water/balloon system. However, the same physical forces that act upon the water/balloon system are fundamental components of the stress state imposed on the tissues of the human body. However, in that case, they are acting in concert with additional forces that have been neglected so far in this discussion. The next section builds on these principles to create an additional layer of sophistication that brings this simplistic model one step closer to the real world.

### 3.3. Human Beings in Space and the Cephalic Fluid Shift

This section is focused on the redistribution of bodily fluids caused by entry into microgravity. Although the conceptual model in this section considers only water for simplicity, it is also applicable for any bodily fluid such as blood, plasma, cerebrospinal fluid, lymph and interstitial fluids. Some background on measurements in actual and simulated microgravity is introduced in Section 3.3.1, alongside an engineering perspective. Other deformable tissues, such as muscle and fat, could also be affected by gravitational change, either at the tissue or organ level, and this less-studied topic will be discussed briefly in Section 3.3.2, along with other important experimental data on fluid shift in space.

#### 3.3.1. Hydrostatic and Biomechanical Effects on Humans in Spaceflight and Simulated Spaceflight

Unequivocal measurements of human physiology in microgravity are difficult to obtain because of small sample size, unique individual responses to space such as motion sickness, differing diets, caloric intakes and energy expenditures, demographic variation, limited measurement capabilities, mission operations and priorities, and variable use of countermeasures such as fluid loading, lower body negative pressure exposure, and exercise protocols. In addition to these confounding factors, the medical data of any American astronaut is subject to the same level of confidentiality as any patient in the United States [67]. As a result, in a desire to protect patient confidentiality, almost all studies provide pooled data rather than individual data, which may mask the effects of interest or hinder subsequent analysis in this small population.

Acute changes imposed by microgravity on humans can be studied during the roughly 20 s provided by parabolic flight, such as precipitous changes in central venous [68] and ocular pressures [20] and choroidal blood flow [69]. Other changes occur much more slowly and the effects may not be evident until after weeks or months of microgravity exposure, such as loss of bone [2] and muscle [3] mass. For parabolic flight, the short periods of microgravity are separated by periods of hypergravity (~2 *g_e_*). We also note that when astronauts are launched into orbit, they undergo about two minutes of hypergravity with a maximum of 2.5 to 3 *g_e_* [45,46]. If the characteristic response time required is short enough, parabolic flights are an excellent laboratory for studying the effect of gravitational changes [53].

In an effort to provide a more controlled environment to study longer-term effects, ground-based simulation of microgravity can be found in studies using bedrest or water immersion. The most commonly used analog of fluid shift is bedrest at a negative incline to promote headward fluid flow (Head-Down Tilt or HDT) [70]. The most common tilt angle used is −6°, at which many of the physiological responses are similar to that of spaceflight. HDT studies don’t mimic launch accelerations, the stressful, tightly scheduled work environment, and they can’t remove the hydrostatic pressure gradient. However, they can redirect the action of the hydrostatic pressure gradient, and their more precise measurements in a controlled environment are a valuable supplement to our understanding of fluid shift [8,70,71,72].

**Figure 9:**
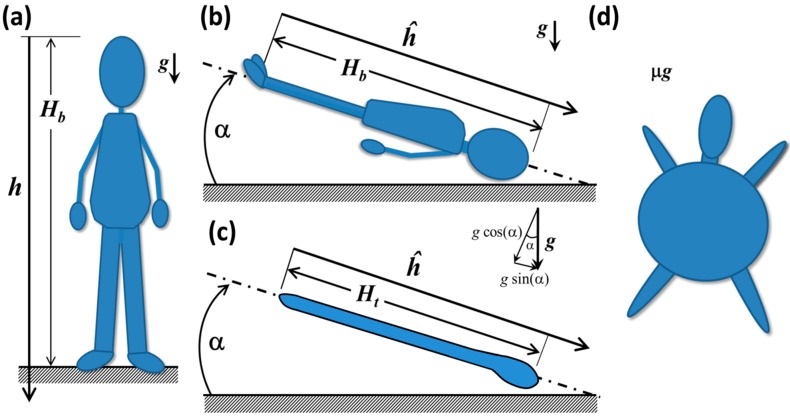
(**a**) Schematic of a hypothetical human-shaped balloon filled with water in an erect posture; and (**b**) in Head-Down Tilt; (**c**) tube-shaped balloon in HDT; (**d**) human-shaped balloon in microgravity.

In this section, we introduce a series of conceptual models that illuminate some of the key physical principles involved in fluid shift. For background on the principles of hydrostatic analysis, e.g., [57]. Consider a human-shaped water balloon, shown in Figure 9a. As is the convention in hydrostatics, the origin of the axis *h* is placed at the upper boundary of the fluid and is positive downward [57]. In reality, the elevation of the reference position is not particularly important because relative pressure *differences* apply hydrostatic force, not absolute pressure.

The hydrostatic pressure difference Δ*p_h_* between the head and any depth along the axis *h* (or any other vertical line that goes through a continuous fluid) can be computed as Δ*p_h_* = ρ*gh,* where ρ is the fluid density, *g* is the gravitational acceleration and *h* is the local depth from the reference location [57], in this case, the top of the head, as shown in Figure 9a. At the balloon’s feet, the hydrostatic pressure difference Δ*p_h_* = ρ*gH_b_* is at a maximum, and it decreases with elevation until it reaches zero at the tip of the head. The magnitude of the impulsive change in hydrostatic pressure after insertion into orbit is directly proportional to the height, *H_b_*, predicting that, if all other factors are equal, the magnitude of the fluid shift would tend to be greater for taller people.

In HDT at some angle α, shown in Figure 9b, some of the fluid in the mock legs and waist has been displaced to the upper body and head due to forces arising from gravity and the balloon’s elasticity. The weight of the displaced fluid creates a hydrostatic force in the region of the balloon’s head that is larger than in the erect human balloon in Figure 9a. In effect, water from the balloon’s legs is pressing against the membranes that encapsulate the upper body and head. The gravitational force is still acting downward in the absolute frame of reference, but the axis of the body, *ĥ*, is no longer aligned with it. From the perspective of the body in the inclined system, the gravity is acting in the cephalic direction [43]. In Figure 9c, a simpler tubular balloon is shown that distends more easily in the longitudinal direction than in the radial direction. This system is closer to the conceptual model typically used to calculate hydrostatic pressures Δ*p_h_* [73] in the arteries, veins and interstitial fluids along the height of the body. Vector addition shows that the magnitude of *g* acting along either balloon axis in Figure 9b,c is *g*ˑsin(α), so that the apparent hydrostatic pressure is ρ*gĥ* sin(α) acting in the cephalic direction [43]. There is also a hydrostatic pressure component acting perpendicular to *ĥ* of magnitude ρ*gĥ* cos(α) acting from the front to the back of the body [57]. If α and the balloon diameter are small enough, this value is close to the actual hydrostatic pressure at the back surface. To examine anteroposterior effects of hydrostatic pressure in the human-shaped balloon, these conditions are violated, particularly in the upper chest. In addition, there is a marked Δ*p_h_* variation along the back of the body due to regional variations in diameter. These remnants of hydrostatic pressure in this analog environment impose a different biomechanical stress state in the body than would be seen in microgravity, in which there would be no hydrostatic pressure.

**Figure 10:**
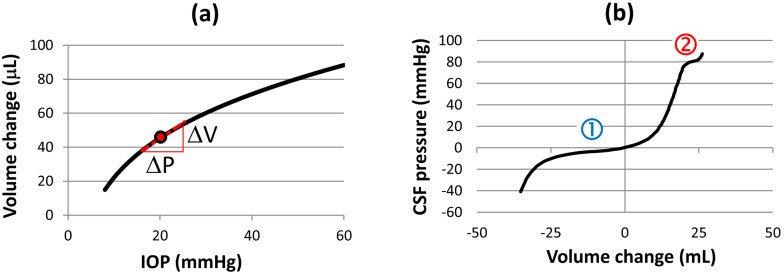
(**a**) Pressure/volume relation for the human eye, adapted from [75]; and (**b**) Volume/pressure relation for the entire cerebrospinal fluid space, adapted from [74].

The system responds to the new loading environment imposed by HDT through the redistribution of fluid and other tissues along the body. In the balloon, the legs, arms, torso and the head and neck can be considered subunits or compartments that are in fluidic communication and can exchange fluid via pressure gradients. The volume change of fluid in a given compartment is related to the dynamic pressure change through the compliance, which is defined as the rate of volume change with respect to pressure, *dV/dp* (e.g., [74]). In Figure 10a, the equilibrium pressure/volume relation for the human eye is shown, as obtained experimentally from injection of saline into the anterior chamber [75]. The compliance, *C*, at a specific *IOP* is the slope of the tangent to the curve and can be approximated by *C* = ∆*V*/Δ*P*, as shown in Figure 10a. The globe volume depends on *IOP* in a logarithmic fashion, so that compliance continuously decreases as pressure increases [75]. One physical interpretation of this curve is that fluid expansion in the globe stretches ocular tissues such as the sclera; the capacity of ocular tissues to accommodate additional fluid declines as the expansion proceeds due to their finite capacity.

Other systems exhibit more nonlinearity, such as the response to fluid injection via spinal tap into the central nervous system [74], shown in Figure 10b. Note that Figure 10b shows pressure as a function of volume, rather than vice versa as in Figure 10a, since it is easier to intuit the physiological behavior in this case. The compliance is still defined as Δ*V*/Δ*P* so that it is the inverse of the slope in Figure 10b. There is significant buffering capacity provided by the much larger reservoirs of arterial and venous blood and by the compliance of the spinal theca, which holds about 2/3 of the overall craniospinal compliance in the spaces filled with CSF [76]. The CSF pressure increases very slowly as fluid is injected near the resting CSF pressure (region 1 in Figure 10b). The volume of fluid in the spinal theca increases more rapidly than the fluid volume in the cranial CSF space due to its greater compliance [77]. In turn, the venous compartment acts as a buffer for changes to CSF volume by undergoing small contractions or expansions [74]. However, the venous system’s ability to perform in this capacity is not unlimited, and the system compliance is reduced substantially at about Δ*V* = 20 mL (as plotted in this figure, compliance is 1/slope of the tangent). As more fluid volume is added, the pressure increases sharply until it approaches the diastolic blood pressure. In region 2, the arterial compartment acts as a buffer for a small CSF volume increase [74].

In microgravity, the human-shaped balloon may resemble Figure 9d, in which the fluid is driven to expand in every direction to the most radially symmetric shape that it is permitted by the balloon, somewhat analogous to the water droplet in Figure 8a. As for the elastic membrane itself, the membrane surfaces would seek a configuration of minimum energy that accommodates the volume of water within it [78]. In HDT, the membrane of the head would be experiencing primarily tensile forces outward in the radial direction as the fluid pushes down upon it. However, the membrane surrounding the legs would be under significant uniaxial tension in the direction *ĥ*. In other words, if the feet remain in a fixed location, those surfaces would be encouraged to stretch the membranes of the legs in the cephalic direction as the fluid presses down toward the head. To determine whether or not these forces are *significant* components of the human physiological response to HDT, additional forces would have to be included in the conceptual model, since it is the sum of the relevant forces in the system that evoke a response.

Since the balloons discussed above were filled only with water, the fluid redistribution would occur almost immediately upon the change in hydrostatic pressure, as discussed in Section 3.1. If the balloons were instead filled with a water-saturated porous medium, the equilibrium balloon shapes might not change, but the time required to attain that shape might be longer and would depend on the time needed for fluid filtration. That time scale would be a function of the geometric properties of the porous medium, such as porosity, pore size and tortuosity, as well as on the material properties of the porous medium and the fluid [79,80]. If there were internal boundaries within the balloon, such as a semipermeable membrane that separated fluid volumes, the presence of other forces, such as osmotic pressure gradients, could also influence the speed of filtration [80]. The addition of differential composition and structure within the balloon would serve to bring the conceptual model another step closer to reality. It could be used to explore adaptation to gravitational changes over longer periods of time.

In these simple engineering models, the local hydrostatic pressure within the balloons could be calculated with precision, since they contained a single continuous fluid. Calculation of local pressure and stress would be more complicated if the balloons contained a multicomponent internal structure more analogous to the actual human body [80]. It is important to note that hydrostatic pressure change could influence any of the deformable tissues in the body, e.g., blood vessel walls [81,82] and the heart [83,84]. In some scenarios, it might be necessary to include the combined effects of surrounding tissues to model the local biomechanical stress state [84], e.g., the organs in the upper chest in HDT. Numerical models, which are based on conceptual models, have begun to incorporate some of the features relevant to fluid shift [85,86,87,88,89,90,91,92], but there is much that remains to be done.

The physical considerations discussed above necessitate that the initial water content of the body, its distribution of fluid and tissues, and the water storage capacity and compliance of bodily tissues will affect the magnitude of the fluid redistribution, both regionally and in total, particularly in the earliest stages. Physics predicts that the equilibrium distribution in the acute to moderate stages of fluid shift will be dependent on:

- The height of the fluid column,
- The initial fluid volume,
- The volume, saturation potential and material properties of fat, muscle and other porous tissues,
- The bones, cartilage and other tissues that may limit volumetric expansion,
- The water storage capacity and compliance of vascular and extravascular systems,
- The presence of semipermeable membranes that separate fluid volumes, such as the blood/brain barrier, and
- The distribution of all these components along the height of the body.

Consequently, physiological attributes which are indicative of the body’s fluid and tissue distribution at a homeostatic 1 g condition, such as body size and type, fitness level, [93] and gender [94], are likely to play a role in the impetus for redistribution in microgravity.

The simple conceptual models described here are best used for insight into the homeostatic distribution of fluids passively driven by changes to the hydrostatic pressure gradient. There are, of course, many more considerations in moving from a human-shaped balloon to an actual human, given the wide variety of tissues and systems, the circulation, regulatory responses, and the loading imposed by everyday living. Not surprisingly, there are differences in the physiological response of a human body to HDT and space, such as the presence of particular signaling molecules used for regulation [95]. The body also has the remarkable ability to remodel its tissues. In some cases, is it a response to biomechanical changes to reach a preferred local stress or homeostatic state, although other factors can also produce changes in tissues [96]. Tissue comprised of collagen fibers can alter fiber orientation and distribution in order to best accommodate a new biomechanical stress state in a matter of weeks or months [97]. For example, hypertension can cause arterial walls to thicken [82,98], and it has been suggested that the sclera can change its thickness in response to a chronic change in refractive error or choroidal thickness [99].

In 2001, Arbeille and co-workers published results demonstrating that many physiological responses in HDT are comparable to actual spaceflight [100]. An important exception was that, in space, the diameters of both the femoral and jugular veins increased significantly by about 35%–40%. In HDT, the jugular vein was comparably distended, but the femoral vein decreased in cross-sectional area. The variation in biomechanical stress state as discussed above in the human-shaped balloon example may well play a role in the physiological response of the femoral vein to HDT.

It is premature to draw too many conclusions about human physiological response to HDT from the simple balloon examples described above. However, they do provide some insight and, more importantly, suggest avenues of exploration in developing improved conceptual models that can be used to test hypotheses. This simple engineering description indicates that HDT is very good at recreating bulk cephalic fluid shift as well as a biomechanical stress state that bears some similarities to spaceflight in the head and upper body.

#### 3.3.2. Experimental Data on the Impact of Cephalic Fluid Shift

On earth, the hydrostatic pressure increases by roughly 10 kPa/m [73] between the top of the head and the soles of the feet, using a simple conceptual model as described in the previous section. Upon entry into microgravity, the hydrostatic pressure is abruptly removed from the tissues of the body, causing a net migration of fluid from the legs toward the upper body and head in a cephalic fluid shift [45,46]. Almost immediately, nasal congestion and a feeling of fullness in the head are reported [43]. The forehead and facial tissues swell and the external neck veins become engorged [7]. A timeline of key physiological changes that accompany spaceflight and return to earth can be found in [101].

In addition to the bulk redistribution imposed by fluid shift, organ shape and position can also respond to microgravity very quickly. In parabolic flight, echocardiography showed that the heart becomes more spherical [60,61]. An independent numerical model validated the modified shape and, by comparing microgravity to lunar, Martian and 1 g simulations, predicted that the stress and strain states of the cardiac tissue at end diastole were a function of gravitational level [83]. The distribution of strain (which is related to tissue response) remained comparable across g levels, although the magnitudes changed. Evaluation of the stress state (which is a measure of applied forces) showed that tissue that was in a state of tension at 1 g could change into a region undergoing compression in space [83]. This change in stress state is perhaps most apparent in observations of inflight lower stroke volume, yet conversely a higher cardiac output. Utilizing a simplified computational model of cardiac function, researchers [102] have illustrated that intrapleural (extracardiac) pressure, represented by a decrease in the hydrostatic intrathoracic pressure, explained the observation of paradoxical stroke volume and cardiac output through compounding effects of physiological responses to changes in local hydrostatic states.

Data from Skylab, the Space Shuttle, Mir and the ISS document the effects of fluid shift from the acute phase to the response over a period of months. Within minutes to hours, facial tissues and eyelids swell [47], astronauts experience nasal congestion and often report headaches and back pain [11,103]. The plasma volume (PV) drops significantly by 10%–15% on the first day or two of flight [104] and in head-down tilt studies [104,105]. Some studies indicate that the drop in PV can be attributed solely or primarily to an increase in urinary output, which is supported by compelling evidence in the controlled environment of HDT [104,105]. In flight, some studies find an increase in urinary volume in the early stages of flight [43,106,107] but the results are not universal [108,109]. Some of the microgravity data may be confounded by voluntary dehydration prior to flight, vomiting induced by nausea, decreased fluid and food intake, and other factors. However, the general consensus is that a net fluid loss is typical in microgravity as well as in HDT. After the initial drop, PV appears to reach a new equilibrium and remains low throughout flight and HDT [21,105,110,111,112]. The rapid reduction of PV can reasonably be expected to at least temporarily reduce the blood volume and increase the concentration of red blood cells (RBCs) in the blood as well as proteins, electrolytes, and other molecular constituents until regulatory or other processes are given time to make adjustments.

**Figure 11:**
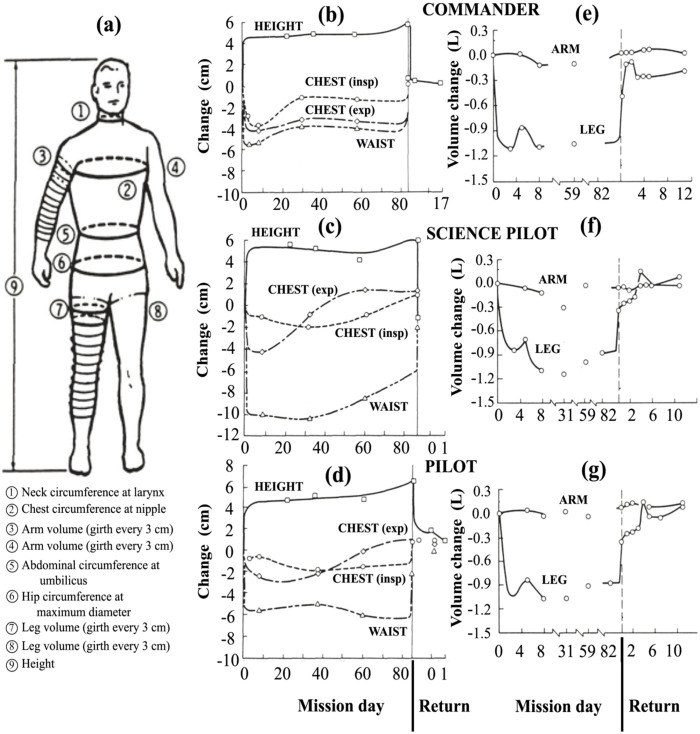
Effect of microgravity on fluid distribution in the human body during the 84-day Skylab 4 mission. (**a**) Measurement locations. The second column shows the change in height and circumference of the waist and chest on inspiration (insp) and expiration (exp) for the (**b**) commander; (**c**) science pilot; and (**d**) pilot. The third column shows left limb net volume change for the (**e**) commander; (**f**) science pilot; and (**g**) pilot. After Thornton [43].

Perhaps the most comprehensive data on the cephalic fluid shift was acquired on Skylab [43]. On three Skylab missions of duration 24–84 days, height was measured and circumferential measurements were taken at the waist, hip, chest, neck and at 3-cm increments along the arms and legs, as shown in Figure 11a. Measurements of body mass for the nine astronauts on these missions showed that there was a net decrease in body mass during flight, which was reversible upon return to 1 g. Thornton attributed the decrease in mass in large part to diuresis and decreased thirst during flight [43]. This study and others have suggested that the loss of body mass can be minimized by matching the caloric intake to the energy output [113,114].

All three astronauts on the Skylab 4 mission exhibited a fairly rapid increase in height of 4 to 6 cm and a decrease of up to 10 cm in the waistline, as shown in Figure 11b–d, as well as a reduction of spinal curvature [43]. Later studies confirmed that the increase in height was almost entirely due to spinal elongation [115,116]. Within the spinal theca, the intervertebral discs swell and increase in height upon gravitational unloading [117]. Thornton suggests that spinal elongation is limited by the anterior spinal ligament [118].

Relative to 1 g supine preflight measurements, the leg volumes of the Skylab astronauts dramatically decreased by about 1000 mL per leg within 4–6 h (see left leg profiles of Figure 11e–g). These figures also show that the arms did not exhibit significant changes in volume, a finding that was confirmed in later Shuttle flights [10,119]. The body’s center of mass shifted 3 to 4 cm in the cephalic direction [43], too much to be explained by the change in height alone. Simultaneous decreases in body mass might have accounted for at maximum about half of the loss in leg volume [43], meaning that at minimum of 1000 mL in volume was shifted from both legs toward the head and upper body. Data from a subsequent Shuttle mission showed similar volume losses in the legs, with about 20% more fluid loss in the non-dominant leg [119]. In addition, the 900-mL volume decrease of the dominant leg was 90% complete within 150 min. On a percentage basis, the thigh lost more volume than the calf since it has more tissue capable of water retention [44].

The volume change in the leg was reversible. Upon landing, 65% of the leg volume was recovered within 1.5 h [43]. Muscle atrophy probably limited the extent of the recovery, since atrophy has been observed in astronauts in just five days [120] unless effective countermeasures were performed. For the Skylab 4 astronauts, the leg volume had returned to preflight values by one month after flight [43]. Similar, but often more limited, fluid redistribution is observed in HDT studies [105,121]. Apparently, the quick fluid volume changes in the legs in response to a change in *g* represent a reversible change in body tissue distribution.

The fast changes to fluid distribution in the body provoke a variety of physiological responses within the first hours or days of flight. Cortisol and antidiuretic hormone levels were found to spike in the blood, indicating that renal and parasympathetic regulatory functions were actively responding to the fluid shift [122]. In HDT, the loss of PV was accompanied by an increase in plasma osmotic pressure by 4 h, likely due to increased protein concentration [123]. Plasma osmotic pressure was also elevated at 14 days of HDT [112]. Relative to preflight values, the blood volume decreased while the hematocrit increased at 22 h and these differences were maintained at day seven [124]. Serum erythropoietin levels decreased significantly in space by the second day in flight, followed by a decrease in red blood cell (RBC) generation over the subsequent week or two [124,125,126,127]. At landing on day 14, both PV and hematocrit were decreased relative to preflight values [124], suggesting that active control of RBC mass had occurred. For a comprehensive discussion of astronaut RBC regulation in space, see [128].

Reports of back pain appear to coincide with the time history of spinal elongation in space. At entry into microgravity, Skylab astronauts determined that there was an immediate elongation of the spine. In the first week or two in space, the spine elongates by about 4–6 cm [10,43,103,115,116] in a roughly exponential fashion [43]. An exhaustive retrospective study of back pain indicates that over half of all astronauts succumb to it during the early stages of flight [129]. The incidence of back pain decreases with time in space [103,129], with exponential decay through day 12 [129]. During this time, the intervertebral discs (IVDs) swell to a level beyond that experienced under routine gravitational loading in 1 g [117]. This may account for the temporary relief of back pain through adopting the knee-to-chest position, which provides compressive loading of the IVDs, thereby reducing disc volume [117]. In HDT, spinal elongation of ~2 cm was noted by day three and it remained at this level through the end of the 16-day HDT study [130]. Back pain increased through days two and three but was not reported by the end of the study [130]. While the time course during HDT and spaceflight may differ, it appears that back pain dissipates as spinal elongation becomes complete.

The apparent gravitational force in 6° HDT (the most commonly used angle in such studies [7]) is roughly 10% of that experienced by a standing human, acting in the direction of the head. This compressive hydrostatic loading in the head and upper body would tend to drive the spinal length to decrease. In contrast, spinal elongation has been observed in three-day 6° HDT [130,131], smaller in magnitude but analogous to what has been seen in spaceflight. In an actual human system, there are other driving forces aside from hydrostatics that that must be considered. One significant force is related to the displacement of CSF from the cranium into the spinal theca in HDT and microgravity. For the cranial fluids as a whole, the capacity to accommodate excess fluid is severely limited by the hard casing of the skull. Upon entry into the reduced gravitational state, there is a rapid flux of fluid that migrates headward. The force imposed by this fluid flux is difficult to estimate *a priori* without a detailed engineering analysis, but it is primarily dependent on the compliances, pressures and volumes of the cranial blood vessels, the brain, and the craniospinal CSF spaces. Tain *et al*. [76] showed that the spinal canal is responsible for about 2/3 of the overall compliance of the craniospinal CSF system. The effect of rapid fluid inflow toward the head caused by HDT was studied by Caprihan *et al*. [132], who examined MRIs of subjects who remained in acute 13° HDT for 40–60 min. The images were studied for HDT-induced modifications to the local gray-scale intensity, which is proportional to water content. The images revealed a 21% drop in intensity in the subarachnoid cerebrospinal fluid compartment, an 11% drop in ocular tissues, and no change in other brain tissues that are associated with cerebral edema [132]. The authors reasonably concluded that cranial blood volume had increased and that some of the CSF within the subarachnoid and ocular spaces had migrated outside the cranium. Likely recipients of the excess fluid volume in the upper body would be the more compliant spinal theca [76] and extracranial venous circulation [86], and possibly the lymphatic system [7]. If the volume of CSF in the spinal theca increases in space, it is possible that the excess fluid could be accommodated through the spinal extension and intervertebral disc swelling observed on-orbit [103,115].

## 4. The Ocular Environment in Spaceflight

In this section, we introduce the evidence of space-related changes on the ocular environment in space due to the effects of chronic alterations to the ocular blood flow, ocular biomechanics and other factors.

### 4.1. Intracranial Pressure Dynamics, Ocular Blood Flow and Biomechanics

The most severe ophthalmological pathologies, as discussed in Section 2, manifested themselves as deficits to visual acuity in most of the affected crewmembers [16]. As seen in Section 3.3.2, the human body reaches an equilibrium with respect to fluid distribution within roughly two weeks, which can be described as a homeostatic state. When visual acuity decrements were reported in the case studies described in Mader *et al*. [16], the astronauts had been aboard the International Space Station for three weeks to three months. At this time, the biomechanical stresses on the ocular tissues associated with acute microgravity exposure had been supplanted for two weeks or more by the chronic homeostatic state in space. Supporting evidence of the role of the homeostatic state includes the inability to produce visual decrements in head-down tilt studies of 2–14 days [16,133], although this is by no means conclusive. On the other hand, repeated flights, even of relatively short duration, appear to be correlated to the occurrence of the Visual Impairment and Intracranial Pressure syndrome [7]. Although this may be confounded with the loss of elasticity associated with increased age [134], it suggests that spaceflight may initiate some as yet unknown mechanism that predisposes the ocular tissues to the Visual Impairment and Intracranial Pressure syndrome even after short missions.

Increased intracranial pressure, venous congestion, choroidal engorgement and vascular or regulatory dysfunction have been cited as potential factors in the development of VIIP [7]. CSF pressure is transmitted to the lamina cribrosa by the cerebrospinal fluid in the retrobulbar space adjacent to the globe posterior, which is at a pressure *p_csf_* [135]. The translaminar pressure difference across the lamina cribrosa (*IOP*-*p_csf_*) can elicit a biomechanical response of the sclera, e.g., foveal displacement [16] or changes to tissue properties [136]. Since it is the pressure *difference* rather than the pressure on either side of the LC that is germane, an increase in *ICP* may not necessarily result in visual acuity changes if the intraocular pressure undergoes simultaneous increases [14,137,138,139,140]. Pressures in fluidic systems, such as *IOP* and *p_csf_*, are affected by the hydrodynamics of fluid flow [141] in the vasculature and CSF space.

While we currently lack quantitative data on *ICP* on humans in long-duration spaceflight, the available data does provide clues. *ICP* increased significantly in the acute response to Head-Down Tilt [142], which is a microgravity analog environment. As discussed in Section 3, acute exposure to microgravity induces a major disruption to fluid distribution and results in increased fluid pressure in the tissues of the head. After an initial spurt in height and a high incidence of back pain, the body gradually adapts to space by elongating the spine and other dispersive mechanisms, with a concurrent reduction in complaints of back pain. Increased *ICP* is consistent with the presence of headaches, e.g., in idiopathic intracranial hypertension [16,143,144], although there are many factors in launch and early adaptation to space that could also be involved. Vein *et al*. [145] concluded that spaceflight triggered headaches, even without space motion sickness, in “super-healthy” men. In another study, HDT was identified as the cause of headache episodes, particularly on day one [146]. All of the data presented above supports the hypothesis that *ICP* spikes initially upon entry into space and may then undergo a relaxation process, although it may not necessarily recover to the 1 g baseline. More direct observations have been made with animal studies. Rabbits in 45° HDT show that *ICP* spikes at the start of the test and decreases exponentially through day eight, at which point they are slightly elevated above baseline [147].

Cerebral hemodynamics should be expected to have significant impact on blood flow throughout the cranium, including the ocular tissues. Both the cranial subarachnoid space and the ocular tissues exhibited reduced water content during HDT, most likely because of increased cranial blood volume [132]. The purpose of cerebral autoregulation is to maintain a constant blood flow to the brain by manipulating the vascular tone and caliber (internal diameter) of cerebral vessels. This occurs through a variety of mechanisms that are not yet fully understood [148]. There is no conclusive literature regarding the impact of spaceflight on cerebral blood flow regulation, but the limited, sometimes conflicting data and associated nuances are covered well in [12]. Rabbits in HDT may again provide some insight. Visual examination of rabbit brains reveal that their external cerebral blood vessels become highly distended and full by day two, a condition which remains through day eight, although to a much lesser degree [147]. Furthermore, no cerebral edema was observed by the researchers. If rabbits, with their simpler cerebral regulation strategies, are able to reduce congestion in the cerebral blood vessels in a week, it is not unreasonable to suspect that humans are also capable of doing so, at least to some extent.

The internal jugular veins are the primary outflow pathway when the body is prone or supine, although differing patterns of cerebral drainage in human beings have been identified [149]. When humans are upright, the jugular vessels collapse due to a drop in hydrostatic pressure [150] and the vertebral and spinal epidural veins provide alternate pathways for drainage [151].

The venous system has, in general, received much less attention than the arterial system, but dysfunctional cerebral drainage could have major implications for the ocular tissues. For example, cerebral venous outflow impairment due to stenosis or compression of the internal jugular veins has been implicated in the development of monocular blindness [152]. Although even recent work describes the cerebrospinal venous system as valveless, there appears to be evidence of valving in the internal jugular vein in children [153] and adults [151,154,155,156,157], even though considerable anatomic variation is present [158]. Some studies have also described the existence of valves in small-diameter vessels of less than 2 mm [159,160].

The discovery of paravenous pathways in the brain [161] and connections of CSF to olfactory lymphatics [162,163,164] could have important implications for VIIP. The lymphatic system may provide another avenue for fluid drainage, thus relieving pressure (*ICP* and/or CSF pressure in the retrobulbar subarachnoid space behind the globe). At this time, a detailed physical description of the lymphatics is the limiting factor in formulating a cohesive numerical model to examine this phenomenon quantitatively [165].

Postural changes are known to produce an immediate effect on ocular tissues and can be used to infer sensitivity to gravity. Aqueous formation [166] and outflow facility [167] appear to be relatively insensitive to gravitational changes, so that an acute increase in blood volume is likely to be responsible for producing any gravitationally induced increase in *IOP* [16]. A postural change from upright to supine produces a relative increase in fluid pressure at the level of the eye, with a corresponding increase in *IOP* [168,169]. In a worst-case scenario, *IOP* was found to double after a fluid pressure increase via a head down inverted posture [170,171]. The relationship between changes to ocular volume and *IOP* has been well studied. When a known volume of saline is injected into the anterior chamber of the eye, the change in ocular volume is related to the increase in *IOP* through the compliance of the sclera and other ocular tissues [172,173].

In parabolic flight, *IOP* increased 58% relative to baseline while the central retinal artery diameter decreased, but not to a statistically significant degree [20]. Preliminary evidence also indicates that *IOP* spikes by 5–7 mm Hg upon entry to space [7]. Mader and co-workers found that *IOP* surged immediately in HDT, but recovered to baseline values by the end of two days [174]. Similar trends in *IOP* response have been confirmed in a variety of HDT studies [21,22,23,24], although there appear to be nuances in the general population. Taibbi and co-workers identified interindividual differences in the pattern of *IOP* change during adaptation to HDT [133]. During acute exposure, Xu *et al*. [24] found that moderate myopes were more sensitive to increases in hydrostatic pressure relative to emmetropes and low myopes.

The choroid is the major supplier of blood to the ocular tissues. Within seconds, choroidal blood velocity increases in parabolic flight and its choroidal volume doubles [69]. Subfoveal choroidal thickness and *IOP* increased during acute (30-minute) 10° HDT, but there was no statistically significant change in retinal thickness [175]. The relevance to ocular function is that a local increase in choroidal thickness at the globe posterior may cause foveal displacement [99], causing a hyperopic shift as well as increased *IOP* [176]. When this important area of research is fortified with additional quantitative data on choroidal response to hydrostatic pressure change, it may be possible to prove or disprove a link between excess choroidal blood volume due to long-term microgravity exposure and anatomical changes to the sclera, such as globe flattening.

Exposure to 3 min of 30° HDT resulted in statistically significant decreases in the diameter of small retinal vessels based on 92 arteriolar locations which exhibited a 3.1% decrease and 86 venular sites which displayed a 3.7% decrease [177]. The resistance to flow, *R*, is extremely sensitive to vessel diameter *d* in that *R* is proportional to 1/*d*^4^. In comparison to a seated posture, inversion caused the IOP and the mean arterial pressure (*MAP*) of the central retinal artery (CRA) to double, while the diameter of the CRA significantly decreased [170] In HDT, the blood velocity in the middle cerebral artery (MCA) was found to decrease by day two, while the diameter of the CRA increased [178]. While this is not conclusive without knowing the corresponding MCA diameter and CRA blood velocity, it raises the possibility that arterial diameter adjusts so that the net blood flow to the brain and ocular tissues remains approximately constant or at least mitigates the most potent disruptions. Adjustments to other blood vessel diameters and velocities are also observed in space, although generally not in tandem. Other studies provide mixed results on the efficacy of many types of regulatory processes in space and HDT [95,179,180,181,182,183,184,185], but the complexity and nuanced nature of this area renders a thorough accounting beyond the scope of this article.

A hydrodynamic perspective can calculate the flows through deformable vessels from first principles such as conservation of momentum, if supplemented by knowledge of the local anatomy (primarily cross-sectional area and branching behavior, if needed), material properties and mean volumetric flowrate or pressures at relevant inlets and exits (e.g., [186,187]). Essentially, a first-principles approach is based on the understanding that the amount of fluid in a domain is equivalent to what was initially there plus what comes into it minus what goes out of it. This approach has been highly successful in modeling the cardiovascular system [188] and *ICP* dynamics [189]. In this type of approach, the rate of nutrient delivery to or waste removal from organs could also be quantified. In extending these insights to ocular flows, it is critical to understand that “blood flow” (as measured, e.g., in μL/min) is not equivalent to “blood velocity” (e.g., in cm/s) [190], and knowledge of the pulsatile characteristics of the flow does not quantify the amount of blood that flows to or from particular organs. Access to retinal vessels by precise techniques such as laser Doppler velocimetry and high-resolution visual imagery is relatively straightforward. Such combinations have allowed measurements of volumetric flowrates in individual retinal arteries [191,192] and veins [192] and, more importantly, net retinal throughput [193,194].

In a study with open angle glaucoma patients and healthy controls, Feke and co-workers [195] measured the mean values for diameter and velocity, and calculated flowrate at a selected site in the inferior temporal retinal artery after 15 min of sitting. Baseline mean values were comparable in each group. Postural change to reclined rest induced large variations in vessel diameter and velocity, but since the mean cross-sectional area *A* decreased proportionally to the increase in mean velocity *V*, the mean flowrate *Q* (which is the product of *A* and *V*), was essentially constant in healthy controls. On the other hand, glaucoma patients exhibited three different patterns of response, which may provide useful diagnostic information as to the source of dysfunction and decisions for treatment.

In a larger study of 64 adults, Garhöfer *et al*. [193] measured a net retinal blood flow of 44.0 ± 13.3 uL/min by summing flowrate contributions from all retinal arteries larger than 60 μm entering the optic nerve head. They then separately summed the blood flows through all of the arterioles and all of the venules. All three summations exhibited comparable throughput, as would be expected when the key components of net inflow and outflow are captured. Other groups have reported somewhat different flowrates, but the measurements in this study were comparable to within measurement error with Garhöfer *et al*.’s prior measurements, suggesting that they obtained an internal consistency within measurements in their lab.

These studies provide support that retinal regulation is targeted at providing the proper nourishment to the retina, which is primarily a function of net flowrate, and acts through mediating influences such as modification of vessel cross-sectional area and vascular tone. Simultaneous measurements of *V* and *A* (or *d* in vessels with circular cross-section) permit calculation of *Q*. The availability of all three can provide a richer understanding of retinal hemodynamics and permit different kinds of comparisons between individuals and populations.

Similar quantitative evidence does not appear to exist for the vasculature of the choroid nor for the ocular tissues as a whole. Ambarki *et al.* [196] recently measured the mean flowrate in the ophthalmic artery (OA) as approximately 10 mL/min through the use of phase contrast MRI. The pulsatility of the flow varied significantly between healthy young and healthy elderly subjects. Interestingly, *Q* was statistically identical in both populations. As a rule of thumb, about 25% of the blood flow in the OA is routed to ocular tissues [197]. The OA branches into several vessels that terminate at the ocular tissues: two anterior ciliary arteries, a retinal artery and four or five posterior ciliary arterial (PCA) trunks, which in turn branch into 10–20 short PCAs at entry into the globe and two long PCAs. About 85% of that ocular blood nourishes the choroid while 15% supplies the retina [198]. These arteries then branch into successively smaller arterioles and the capillary bed to supply the ocular tissue with nutrients.

The bulk of the ocular blood leaves the eye via the “vortex veins” (VVs). They receive the name by virtue of the spatially rapid collapse of many smaller vessels into a large ampulla from which a single vein emanates. The vessel walls on either side of the ampulla are densely packed with smooth muscle cells, while the thin-walled ampulla is comprised of a single layer of endothelial cells [199]. The VVs merge with the superior and inferior ophthalmic veins (OVs).

**Figure 12:**
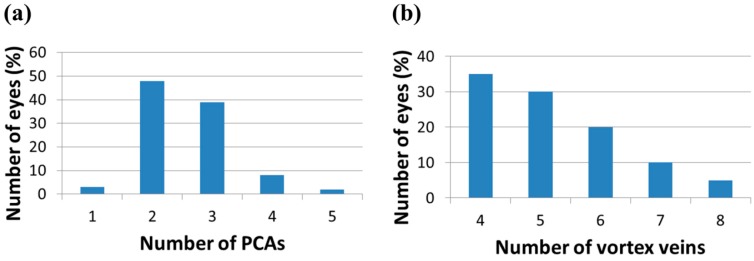
Number of major blood vessels in choroidal (**a**) inflow (data obtained from [202]) and (**b**) outflow (data obtained from [204]).

Theoretically, measurements of net choroidal flow could be made by summing contributions from the PCA trunks to characterize inflow and the VVs for outflow. Efforts to comprehensively sample the blood velocities in the various ocular vessels have been made (e.g., [200,201]), but it is not typical practice to characterize supplement the mean velocity with measurement of vessel diameters, e.g., with high-resolution fundoscopy, nor to determine net flowrates. The preponderance of studies use color Doppler imaging to measure blood velocity in ocular vessels, but limitations of the technique along with the diminutive size and more challenging access [201] appear to have limited thorough examination of all of the vessels. There are considerable interindividual differences in the number of PCA trunks [202,203], as shown in Figure 12a, and in the number of their daughter short PCAs entering the orbit. Despite the conventional wisdom that there are 4 VVs per orbit, the VVs also differ in number [204,205] (Figure 12b), vessel size and carrying capacity [205]. A measurement of the net ocular inflow and outflow would require an accounting of the flowrates in all of the VVs and all of the PCA trunks (or their daughters) since there is asymmetry in the carrying capacity of individual VVs and PCAs. One benefit of knowing the net inflow and outflow is that it would remove the variable of widely disparate individual branching structures and permit interindividual and interpopulation comparison at the level of the choroid as an organ.

The description of valves in the superior OV [206] and the recent discovery of outflow pathways from the ocular surface to nasal mucosa [207] may have profound implications for drainage from the eye. Studies on regulation of the retina (e.g., [177,195,208,209,210,211,212]) and more recently on the choroid [99,210,211,213,214] could be strengthened with more comprehensive measurements of the blood flow to and from these organs.

### 4.2. The Spacecraft Environment and Other Potential Factors in the Development of VIIP

The uniqueness of the spaceflight environment may introduce other factors that play an additive role in the etiology of reduced visual acuity. The particular characteristics of a spacecraft environment are dependent on the mission requirements and the balance of engineering and operational risks. Environmental factors include vehicle atmospheric pressure and component concentrations, radiation shielding and solar particle events. Operational parameters include available daily drinking water, and even the mass and storage of food and medical supplies, which constitute potential tradeoffs with engineering parameters. For instance, the engineering and operational requirements to maintain the ISS lead to:

- Low humidity and high CO_2_ concentrations, with periods of exposure to low total pressure and 100% O_2_ during extravehicular activity,
- Increased radiation levels with the potential of exposure to high energy ‘cosmic ray’ radiation,
- A high salt diet resulting from a change in taste acuity, and
- A disrupted circadian rhythm.

Many of these factors, potentially in combination with microgravity fluid and tissue redistribution and individual characteristics, have been hypothesized to influence the progression of visual acuity loss in astronauts [7].

Since the ISS is a closed environment, respiration and other sources of formation cause a rise in CO_2_ concentrations unless there is atmospheric control. External venting or chemical “scrubbing” are used to remove CO_2_ from the air that the astronauts breathe. Another consequence of microgravity is that naturally occurring atmospheric mixing by gravitational forces is not present, resulting in localized buildup near any CO_2_ source. Until recently, the CO_2_ partial pressure in the ISS environment could reach levels up to 5 mm Hg, or approximately 20 times greater than CO_2_ concentrations at sea level. Terrestrial data does not imply significant acute or long-term effects, even with significantly elevated concentrations [7,215]. Nevertheless, CO_2_ is a powerful vasodilator, and hypercapnia can also increase cerebral blood flow, elevate ICP, and increase the formation of cerebrospinal fluid [216]. On orbit, there are numerous anecdotal reports of headaches and feelings of confinement that have been attributed to CO_2_ toxicity and the inability of the air-handling system to remove CO_2_ from confined spaces in a timely manner. From this evidence, it was hypothesized that chronic elevated CO_2_ exposure, in conjunction with the microgravity-induced fluid shift, could potentially result in an increased susceptibility to the VIIP syndrome [7]. In response to this medical concern, the CO_2_ levels on ISS have been reduced.

The characteristics of an individual’s metabolic pathways in space may also play a role in the development of VIIP. A 2012 study of astronauts with and without the ophthalmic changes of VIIP illustrated an altered metabolic pathway involving homocysteines, cystathionine, 2-methylcitric acid and methylmalonic acid that has been linked to affected crewmembers [217]. In this study, the astronauts who exhibited vision changes demonstrated a preflight and inflight alteration in their folate- and vitamin B-12-dependent 1-carbon transfer metabolism, suggesting that polymorphisms in enzymes of this pathway may interact with the spaceflight environment to negatively alter anatomical and physiological responses. The causality of this increased sensitivity, including genetic and biomechanical influences on the progression, is still under investigation.

Astronaut nutrition during spaceflight is also considered a potential influencing factor in ophthalmic changes. In general, astronauts have excellent health and nutritional status in comparison to the general population, however, the typical ISS diet is relatively high in sodium, approaching 5 g per day [7]. This is attributed to a decrease in taste sensitivity in the spacecraft environment [218]. There has been speculation that sodium negatively influences intracranial pressure and other orthostatic changes in astronauts, although the precise mechanisms have not yet been adequately defined [7]. A nutritional influence on homocysteine levels has been suggested, although high homocysteine levels associated with nutritional deficits have not been observed in the astronaut population [217].

Alterations in the radiation environment leading to acute radiation syndrome or postflight degenerative radiation induced diseases are a significant health risk for astronauts. High-energy solar particle events (SPE) and Galactic Cosmic Rays, ranging from hydrogen nuclei (protons) to high-energy iron nuclei, make up the space radiation environment. Among the potential effects, an impact on circulatory diseases and a reduction in central nervous system function are considered the most deleterious [219], although the potential influence on visual acuity has not been elucidated. A recent preliminary report involving a porcine model study indicated that exposure to simulated SPE radiation resulted in increased *ICP* over the 90-day experimental period [220], which indicates that the role played by radiation in the progression of visual acuity loss is still to be fully described.

Without intervention, the gravitational unloading of the musculoskeletal system leads to significant loss in bone mineral and muscle mass [2]. A primary countermeasure is on-orbit resistive and aerobic exercise. By necessity, to replace gravitational loading, astronaut exercise prescriptions include high resistive loads [221], whose influence on CSF pressure is unknown. Evidence suggests that high resistive exercise does not increase *ICP* if performed without a Valsalva maneuver [7,222] and that *ICP* is reduced after aerobic activity [223]. These effects have important implications on the manner in which exercises should be performed in space, their impact on the progression of fluid redistribution, and, potentially, on the progression of visual acuity changes.

Changes in astronaut sleep patterns may also play a role in changes to *ICP*. Astronauts can experience alterations to their circadian rhythms due to changes in exposure to sunlight and changes in working periods [224], which may manifest in reductions in performance or increased fatigue. A recent study in mice has shown that sufficient sleep increases the exchange of CSF with interstitial fluid by up to 60% [225]. This striking increase in fluid exchange, in combination with the altered sleep patterns of astronauts, implies that further assessment should be made as to the influence of sleep and fluid redistribution during long-duration spaceflight.

Although intriguing, the evidence supporting the overall importance of each of these factors is inconclusive at this time. The analysis is confounded by sparse data and interrelated influences that hinder the determination of the causal chain from a potential contributor to the observed response. It is clear, however, that many factors related to space travel have the potential to alter the fluid redistribution process, as well as CSF pressure and visual acuity.

## 5. Concluding Remarks

Over 50 years of human spaceflight has shown that the human body can adapt to the microgravity environment through many functional changes, including fluid and tissue redistribution, allowing humans to live and work in space for extended periods. Unfortunately, these adaptations can lead to short- and long- term loss of physiological function and increased risk of serious medical conditions. At this time, one of the highest perceived medical risks to long-duration spaceflight is the potentially permanent loss of visual acuity. All of the evidence suggests that the Visual Impairment and Intracranial Pressure syndrome exhibited in astronauts is induced by some combination of factors inherent to microgravity and other factors in spaceflight.

Upon introduction into microgravity, the most obvious effect of spaceflight is the aggressive redistribution of bodily fluids from the legs toward the upper body, including blood, plasma, cerebrospinal fluid, lymph and interstitial fluid. The acute response can be studied within the 20-second intervals of microgravity seen in parabolic flight. The heart becomes more spherical, intraocular pressure spikes, the spine elongates, and the bulk fluid/tissue distribution shifts in the cephalic direction almost immediately. Plasma volume drops sharply and reaches a new equilibrium within about two days. During and after the gross redistribution, regulatory processes, osmotic and transmural pressure gradients and tissue stress gradients continue to refine the compartmental distribution of the fluids. The rearrangement of body mass distribution is essentially complete by the middle to end of the second week of flight. The upper body vasculature, spinal theca, interstitial tissues and possibly the lymphatic system are likely repositories for much of the redistributed fluid. Ground-based experiments suggest that blood displaces some of the cranial CSF, and that intracranial pressure increases in the acute phase. Inflight measurements of *ICP* have not yet become available, however, although this is being actively pursued. *ICP* is important to ocular biomechanics because, assuming that there are no blockages, it sets the pressure in the retrobulbar subarachnoid space behind the eye.

As described in this review, the fluid shift causes the body to reach a new “space-normal” homeostatic state, which is driven by the loss of hydrostatic pressure gradients as encountered on earth. This paper introduced simple water-filled balloons to derive insight into the biomechanical stresses applied on membrane geometry and differing orientation and magnitude of the hydrostatic pressure gradient. It also described the means by which such a model could be further developed by incorporating additional tissues such as muscle and fat. This approach could be used to quantitatively examine the impact of factors associated with the spatial distribution of these tissues, including body size and type, gender, BMI, and fitness level. In order to develop a meaningful model, quantitative measurements of tissue material properties and their spatial distribution would also be needed, both to build the model and to validate its predictions.

Cerebral and ocular hemodynamics are likely to play a significant role in the biomechanical stress state imposed on ocular tissues in space. The volume of blood in the ocular tissues has a direct impact on the *IOP*, which would in turn affect the translaminar pressure gradient. The availability of drainage pathways, the compliances of the tissues of the venous, arterial, lymphatic and central nervous systems, and the role of regulation are essential factors to the understanding of blood flow within the cranium. The retinal tissues appear to be governed by effective regulation in gravitationally related fluid shifts in healthy people, but less is known about the response of the choroid.

All of the tissues in the human body are subjected to a substantially different set of biomechanical stresses in the spacecraft environment, and they may be in excess of those routinely encountered on earth. Over the course of many weeks or months, some tissues, such as the sclera, have the capacity to remodel in order to realign their biomechanical properties with the new homeostatic state. A better understanding of the relationship between the biomechanical stress state in the eye and tissue remodeling could provide the link between biomechanics and some of the anatomical changes observed in VIIP. In addition to biomechanical responses, these tissues may have increased sensitivity to particular metabolic pathways and environmental factors, leading to increased susceptibility for unfavorable adaptations, such as degraded visual acuity, that are observed in the VIIP syndrome.

The cephalic fluid redistribution causes dramatic physiological changes upon entry into microgravity and it is present throughout the mission. Consequently, it is inextricably linked to a cascade of physiological responses to space, which have yet to be fully described. Although no complete pathway from exposure to space to VIIP can yet be determined, the profound impact of fluid shift must be considered as one of the more likely contributors to the condition. It is likely to act in concert with other potential contributing factors such as diet, radiation exposure, atmospheric composition, genetic and physiological or anatomical predispositions.

At present, it is unclear if the reduction in visual acuity is progressive with time on orbit or if most of the persistent effects occur early in spaceflight. The level of uncertainty in understanding this condition is relatively high, but that understanding is critical to continued success in long-term spaceflight. The NASA Human Research Program is actively engaged in studying the initiation and progression of the VIIP syndrome through studies on the International Space Station and on the ground, including HDT studies and numerical investigations, as well as the development of new on-orbit diagnostic hardware. Over the next several years, the Human Research Program is poised to make major strides in unraveling the tangled web of processes involved in the etiology of the VIIP syndrome.

## Nomenclature and Abbreviations

*A*: Mean cross-sectional area inside the blood vessel
COP: Colloid Osmotic Pressure
CPG: Clinical Practice Guidelines
CRA: Central Retinal Artery
CSF: Cerebrospinal fluid
CWS: Cotton wool spots
*d*: Diameter
GCR: Galactic Cosmic Rays
*g*: Gravitational or net acceleration
*g_e_*: Gravitational acceleration at sea level on earth
*h*: Axis along which gravity acts
*ĥ*: Local axis aligned with the height of the body
*H*_b_: Total height of the body
*H_t_*: Total height of the tubular balloon
HDT: Head-Down Tilt
*ICP*: Intracranial Pressure
IIH: Idiopathic Intracranial Hypertension
*IOP*: Intraocular Pressure
ISS: International Space Station
*m*: Mass
MCA: Middle Cerebral Artery
MR: Magnetic resonance (imaging)
OCT: Optical coherence tomography
OA: Ophthalmic artery
OD: Oculus dexter (right eye)
ON: Optic nerve
OND: Optic nerve disc
ONS: Optic nerve sheath
ONSD: Optic nerve sheath diameter
OS: Oculus sinister (left eye)
OV: Ophthalmic vein
*p_csf_*: Cerebrospinal fluid pressure
PV: Plasma Volume
*Q*: Mean volumetric flow rate
RBC: Red blood cell
SPE: Solar particle event
SRB: Solid Rocket Boosters
SSME: Space Shuttle Main Engines
*V*: Mean velocity
VIIP: Visual Impairment and Intracranial Pressure
*W*: Weight
Δ*p_h_*: Hydrostatic pressure gradient
ρ: Density
